# Cryosurgery would be An Effective Option for Clinically Localized Prostate Cancer: A Meta-analysis and Systematic Review

**DOI:** 10.1038/srep27490

**Published:** 2016-06-07

**Authors:** Liang Gao, Lu Yang, Shengqiang Qian, Zhuang Tang, Feng Qin, Qiang Wei, Ping Han, Jiuhong Yuan

**Affiliations:** 1 The Andrology Laboratory, West China Hospital, Sichuan University, 37 Guoxue Xiang, Chengdu, Sichuan 610041, China; 2 Department of Urology, Institute of Urology, West China Hospital, Sichuan University, 37 Guoxue Xiang, Chengdu, Sichuan 610041, China

## Abstract

Cryosurgery (CS) has been used on patients with clinically localized PCa for more than 10 years. However, clinical studies evaluating its effectiveness and safety have reported conflicting results. This systematic assessment was performed to obtain comprehensive evidence regarding the potential benefits and safety of CS compared with those of radiotherapy (RT) and radical prostatectomy (RP), respectively. All controlled trials comparing CS with RT or RP and single-arm studies reporting results of CS therapy were identified through comprehensive searches of PubMed, the Cochrane Library and Embase. Ten publications from seven trials, with totally 1252 patients, were included in the meta-analysis, which revealed no significant differences in comparisons of CS vs RT and CS vs RP for overall survival and disease specific survival. However, a significantly lower disease-free survival could be observed for CS than RP. Moreover, a systematic review of literature focusing on comparative data of databases and materials of single-arm trials revealed satisfactory survival results in both primary and salvage CS. Our results showed that cryosurgery would be a relatively effective method for clinically localized prostate cancer with survival results comparable to radiotherapy and radical prostatectomy. However, the large percentage of complications caused by cryosurgery should be carefully monitored.

With the advent of widespread screening of prostate-specific antigen (PSA) testing, increasing number of males have been diagnosed with prostate cancer (PCa). Cancer statistics of the United States estimated that there were about 233,000 newly diagnosed PCa patients in 2014, with 29,480 patients succumbing to the disease, PCa was the most frequent cause for morbidity and the second most common cause for mortality in males^1^.

Traditionally, although multiple choices were available, including watchful waiting/active surveillance, castration, radical prostatectomy (RP), radiotherapy (RT), chemotherapy and minimally invasive treatments, an appropriate treatment choice for PCa has remained uncertain and controversial over a long period^2^. However, considering the deficiencies in radical therapies (RP and RT), minimally invasive treatments have been gradually adopted. To date, several minimally invasive treatments have been applied to PCa, among which ablative CS is the most widely chosen.

CS was first applied into PCa in 1964 and had undergone several modifications^3^, which have made it more effective in both primary and salvage therapy. However, it has been prevalent mainly in the previous two decades. This operation is accomplished by destroying the prostate by inducing the formation of intracellular ice with an extremely low temperature under the guidance of imaging; the freezing triggers cellular apoptosis and the inflammatory cascade^4^. Several studies aimed at evaluating the efficiency and safety of cryosurgery for PCa have been carried out, but have demonstrated conflicting results.

In this study, we aim to obtain more definitive results by evaluating and comparing the potential benefits and complications of CS with those of RP/RT, and comprehensively reviewing the available literature.

## Results

### Meta-analysis

**Figure 1:**
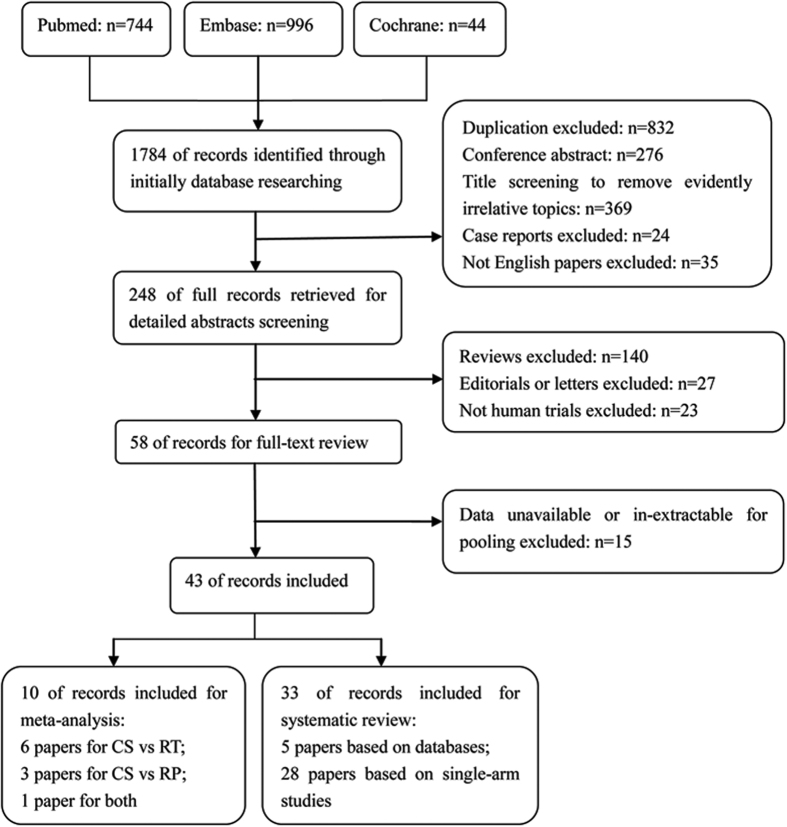
Data flow chart of meta-analysis. CS = cryosurgery; RT = radiotherapy; Rp = radical prostatectomy.

In total, 10 papers from seven studies with 1252 patients (561 for CS vs RT, 745 for CS vs RP and 54 for both) were finally included for pooling in our study^5^,^6^,^7^,^8^,^9^,^10^,^11^,^12^,^13^,^14^ (Fig. 1). The characteristics of each study are summarized in Table 1.

Seven papers^5^,^6^,^7^,^8^,^9^,^10^,^11^ from five studies reported the comparison between CS and RT, from which two articles^7^,^8^ based on one study presented results in mean follow-ups of 37 months and 105.2 months, respectively, results from one study were presented simultaneously in two papers^9^,^10^. Both these studies were RCTs. One study was prospective^5^ and two studies were retrospective^6^,^11^. Three studies were also conducted for primary treatment of PCa, one^11^ for salvage treatment after primary RT and one for both primary and salvage treatments^5^. Only one study had provided available materials for both comparisons^11^.

For comparison between CS and RP, a total of four studies^11^,^12^,^13^,^14^ were involved in the analysis, of which one was prospective whereas three were retrospective. Furthermore, two studies each revealed the results of primary^12^,^14^ and salvage^11^,^13^ procedures. One study^12^ described two CS methods, total and subtotal, which were summed into the CS group, whereas similar summation was used in another study^14^ for the RP group.

### Primary Outcomes

#### Overall survival (OS)

**Figure 2:**
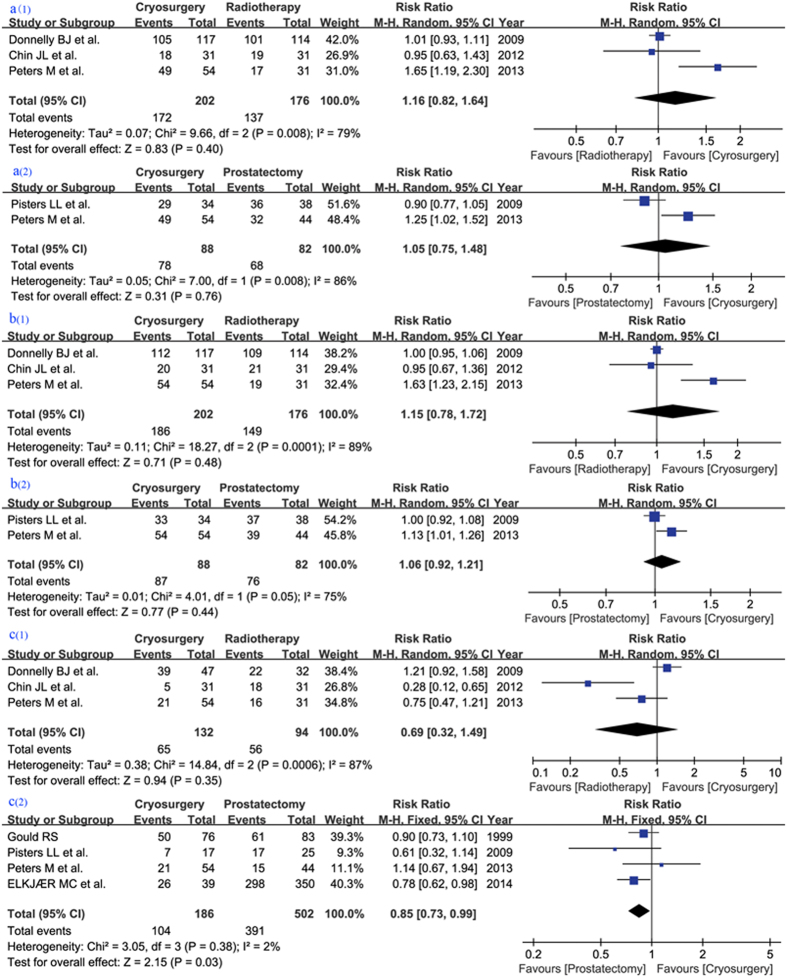
Forest plot and meta-analysis of overall survival (OS) in comparison of cryosurgery (CS) vs. radiotherapy (RT) [a(1)] and CS vs radical prostatectomy (RP) [a(2)]; disease-specific survival (DSS) in comparison of CS vs RT [b(1)] and CS vs RP [b(2)]; disease-free survival (DFS) in comparison of CS vs RT [c(1)] and CS vs. RP [c(2)]. M-H = Mantel-Haenszel test. CI = confidence interval.

Four studies^8^,^10^,^11^,^13^ including a total of 494 patients, three publications^8^,^10^,^11^ with 378 patients evaluating CS vs RT and two^11^,^13^ publications with 170 patients evaluating CS vs RP (including one study^11^ reported both) assessed OS; of these, two studies^11^,^13^ had focused on salvage treatment. Data revealed a tendency for an advantage in the CS group, but insignificant differences (RR 1.16, 95% CI 0.82–1.64, p = 0.40, and RR 1.05, 95% CI 0.75–1.48, p = 0.76, respectively) (Fig. 2a).

#### Disease specific survival (DSS)

These four studies had also reported DSS, which demonstrated similar results to OS (RR 1.15, 95% CI 0.78–1.72, p = 0.48 in CS vs RT and RR 1.06, 95% CI 0.92–1.21, p = 0.44 in CS vs RP) (Fig. 2b).

#### Disease-free survival (DFS)

Data describing DFS were pooled from six studies^8^,^10^,^11^,^12^,^13^,^14^, of which three^8^,^10^,^11^ with 226 patients evaluated CS vs RT whereas four^11^,^12^,^13^,^14^ with 688 cases regarding CS vs RP, including one study^11^ reporting three kinds of treatments. However, comparing OS and DSS, opposite tendencies were presented in both comparisons, in which significant differences could not be found in CS vs RT (RR 0.69, 95% CI 0.32–1.49, p = 0.35) but in CS vs RP (RR 0.85, 95% CI 0.73–0.99, p = 0.03) (Fig. 2c).

### Secondary outcomes

In total, three publications^5^,^6^,^9^ of evaluating CS vs RT had provided available data for pooling, from which only six patients in one study^5^ received salvage cryosurgery. Furthermore, these three publications dynamically (two with continuous variables and one with dichotomous variables) described follow-up in multiple stages: urinary and sexual bothers in months 1, 3, 6, 12 and 24 were extracted for analysis.

#### Urinary bother

**Figure 3:**
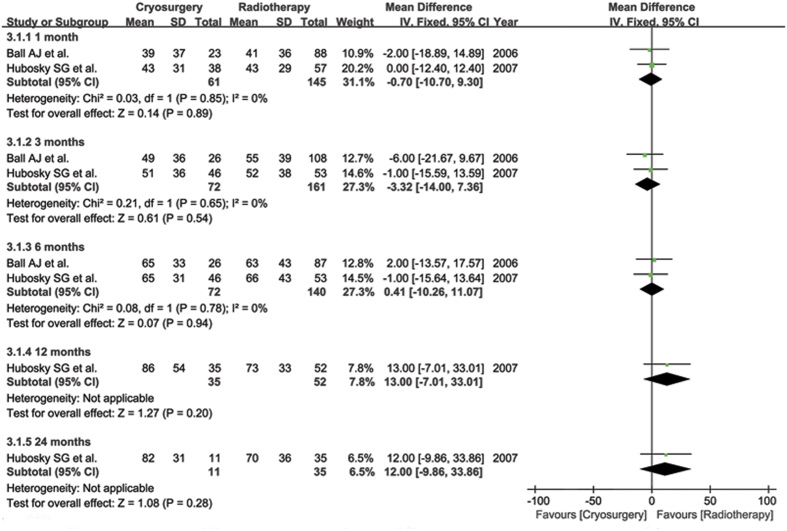
Dynamical forest plot and meta-analysis of University of California Los Angeles, Prostate Cancer Index (UCLA PCI) scores focusing on urinary bother in comparison of CS vs RT in months of 1, 3, 6, 12 and 24, respectively.

**Figure 4:**
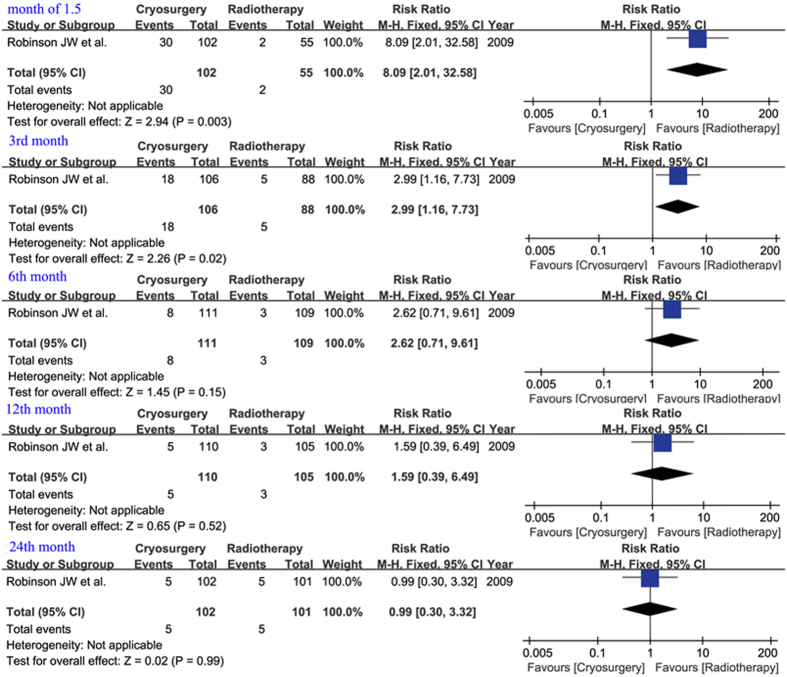
Dynamical forest plot and meta-analysis of patients reporting a moderate or severe problem of urinary bother in comparisons of CS vs RT in months of 1.5, 3, 6, 12 and 24, respectively.

According to UCLA PCI scores, pooled data from continuous variables of two studies^5^,^6^ revealed that there was no significant difference in each stage, but slightly lower scores were observed in the RT group in months 12 and 24 (WMD 13.00, 95% CI −7.01–33.01, p = 0.20 and WMD 12.00, 95% CI −9.86–33.86, p = 0.28, respectively) (Fig. 3). However, differences between 1.5 and 3 months were significant in the dichotomous variable of one study^9^ (RR 8.09, 95% CI 2.01–32.58, p = 0.003; and RR 2.99, 95% CI 1.16–7.73, p = 0.02) (Fig. 4).

#### Sexual bother

**Figure 5:**
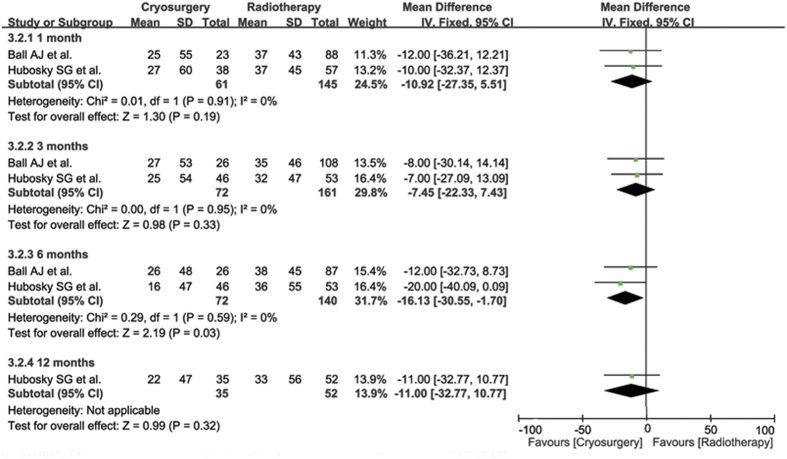
Dynamical forest plot and meta-analysis of UCLA PCI scores focusing on sexual bother in comparisons of CS vs RT in months of 1, 3, 6 and 12, respectively.

Compared with RT group, the CS group presented tendencies of lower UCLA PCI scores for sexual bother at all stages, including a significantly lower score in the six month (WMD −16.13, 95% CI −30.55 to −1.70, p = 0.03) (Fig. 5). Similarly, significantly more sexual bother were presented in the CS group at all stages than the RT group (RR 1.84, 95% CI 1.25–2.73, p = 0.002 in month 1.5; RR 2.25, 95% CI 1.55–3.27, p < 0.0001 at 3 months; RR 1.66, 95% CI 1.20–2.29, p = 0.002 at 6 months; RR 1.68, 95% CI 1.20–2.34, p = 0.002 at 12 months and RR 1.63, 95% CI 1.18–2.26, p = 0.003 at 24 months).

#### Biopsy Positivity and cancer metastasis

Two publications^8^,^10^ reported no significant difference in the number of positive biopsies after primary treatments of CS and RT (RR 0.54, 95% CI 0.13–2.33, p = 0.41). A similar result was found in cancer metastasis (RR 0.67, 95% CI 0.12–3.72, p = 0.64).

#### Sensitivity analysis and publication bias

Only two RCTs regarding CS vs RT were retained for sensitivity analysis, which revealed nearly equal OS (RR 1.0, 95% CI 0.91–1.11, p = 0.96) and DSS (RR 0.99, 95% CI 0.92–1.07, p = 0.86), and equivalent rates of biopsy positivity and metastasis and complications. A trend favoring RT (but without significance) was evident (RR 0.60, 95% CI 0.11–3.21, p = 0.55). No RCT was available for comparing CS vs RP.

### Systematic Review

#### Comparisons from databases

During our searches, five publications^15^,^16^,^17^,^18^,^19^ based on two databases were extracted, of which one study was carried out on the basis of the Cancer of the Prostate Strategic Research Endeavor (CaPSURE) registry whereas the other four studies were from Surveillance Epidemiology and End Results (SEER)-Medicare linked database. Basic information about these studies is summarized in Table 2.

The CaPSURE registry was used by White *et al*.^15^ to evaluate the quality of life in patients with T3 or T4 PCa, and revealed significant decreases in urinary and sexual function according to UCLA PCI scores after treatment. Similar differences in urinary and sexual function could also be found between types of treatment; however, data for further analysis was unavailable.

Four publications^16^,^17^,^18^,^19^ reported outcomes on the basis of the SEER-Medicare linked database, of which three studies^16^,^17^,^19^ concentrated on primary treatments whereas the remaining study^18^ focused on salvage treatments. According to Williams’ study^16^, there were significantly higher overall complications, urinary complications and ED in the CS group than those in the RT group (RR 1.33, 95% CI 1.27–1.40, p < 0.00001 for overall complications; RR 1.77, 95% CI 1.62–1.93, p < 0.00001 for urinary complications; and RR 1.59, 95% CI 1.45–1.75, p < 0.00001 for ED).

In addition, Roberts *et al*.^17^ reported that >28.7% of patients experienced urinary complications and 20.1% ED, after CS. Moreover, in a study by Jarosek *et al*., urinary complication incidence of 19.4%, 19.7% and 27.2% was reported in CS, RT and RP groups, respectively (RR 9.84, 95% CI 8.89–10.89, p < 0.00001 for CS vs RT; RR 0.71, 95% CI 0.65–0.78, p < 0.00001 for CS vs RP) (EBRT, BT and their combination were regarded as the RT group). Only one study^18^ for salvage treatments presented an OS of 93.9% vs 78.4% (p = 0.001) and a DSS of 98.6% vs 93.5% (p = 0.07) for CS vs RP.

Furthermore, two studies^16^,^18^ carried out an economic analysis and revealed significantly lower costs in the CS group than both the RT group and the RP group.

#### Data from single-arm studies

Twenty-eight publications^20^,^21^,^22^,^23^,^24^,^25^,^26^,^27^,^28^,^29^,^30^,^31^,^32^,^33^,^34^,^35^,^36^,^37^,^38^,^39^,^40^,^41^,^42^,^43^,^44^,^45^,^46^,^47^, which had reported availably single-arm results of CS treatment for clinically localized PCa, were screened. Sixteen^23^,^24^,^25^,^26^,^29^,^30^,^31^,^33^,^34^,^36^,^38^,^41^,^42^,^45^,^46^,^47^ of these papers had presented outcomes for primary therapy, eight^20^,^22^,^27^,^28^,^32^,^35^,^40^,^43^ for salvage therapy following RT, one^44^ for salvage therapy following primary CS, and three^21^,^37^,^39^ for both therapies. In addition, materials from the Cryo On-Line Data Registry were used in four publications^30^,^40^,^45^,^46^. Basic information and results of each study are summarized in Table 3.

#### Primary outcomes

Eighteen studies^21^,^22^,^24^,^25^,^28^,^29^,^32^,^34^,^35^,^36^,^37^,^38^,^40^,^41^,^42^,^43^,^46^,^47^ showed the outcomes of OS, which revealed a pooled OS of 73–100% at all stages of follow-up (95.9% at year 1, 94.3 at year 3, 73–98.9% OS at year 5, 91–92% at year 8 and 87% at year 10). Fifteen publications^21^,^22^,^24^,^25^,^27^,^29^,^34^,^35^,^36^,^37^,^38^,^41^,^42^,^43^,^47^ presented results of 79–100% DSS. Differences between primary and salvage treatment were not clear.

Above all, all studies reported the results of DFS, demonstrating results of 25–95.3% for salvage treatments according to the Phoenix definition for recurrence (PSA nadir +2 ng/ml) (83–95.3% at year 1, 72% at year 2, 59–72.4% at year 3, 25–78% at year 5 and 39% at year 8 and 10). However, a higher trend in DFS results could be found for primary studies (44.6–100% overall: 97.9% at year 1, 77.2% at year 2, 82.9% at year 3, 79.6% at year 4, 48–82.9% at year 5 and 89.5% at year 7). In addition, DFS in two studies^33^,^36^ were counted on the basis of only positive results for prostatic biopsy, and demonstrated 84–87.6% DFS up to the end of follow-up. Moreover, three papers reported patients receiving both therapies and revealed 68.8–93.3% DFS.

#### Secondary outcomes

Reports of complications are not easily summarized. (1) For overall complications, one study^36^ reported 19% complications, with 16.7% at grade 3–4 according to the Clavien classification by Kvorning Ternov *et al*.^43^; (2) for urinary complications, including incontinence, urgency, dysfunction, stricture, fistula, etc., a maximum of >53.3%^43^ in total and 41% (increasing) after CS^29^; (3) for ED complications, a maximum of 100%^43^ in total and 56.8% (increasing)^23^. Another study^29^ presented 33.4% (increasing) for level 3 and higher erectile function according to International Index of Erectile Function-5 (IIEF-5) for patients followed with CS. However, Bahn *et al*.^25^ introduced an investigational and focal CS for patients with a strong desire for the preservation of sexual function, which decreased the frequency of irremediable ED to 11.1%.

## Discussion

Over the last few decades, the promotion of screening of PSA for detecting PCa has resulted in a stage migration towards an increase in the occurrence of lower stages, resulting in a controversy regarding the overdetection and overtreatment of PCa. In general, radical therapies, such as RP and radical RT, are regarded as the most effective methods for localized PCa. However, the benefits of these therapies compared with conservative observation remain under debate.

As a compromise between radical treatments and conservation, focal therapy has been applied increasingly as an effective modality for the cure or control of early cancer with minimal injury, especially for patients who cannot tolerate RP or RT. Multiple technologies have been applied for focal therapy, such as CS, high intensity focused ultrasound (HIFU), vascular-targeted photodynamic therapy, etc. Being one of the most popular focal therapies, CS has been applied to a quantity of solid neoplasms and achieved satisfactory outcomes. Four main pathways may have contributed to its mechanism: direct cell injury, vascular injury and ischemia, apoptosis, and immunomodulation^48^.

The results of initial trials have made CS very popular. However, subsequent controversies on its efficiency, safety and applications surfaced. Up to know, only two RCTs have been conducted which revealed conflicting results. To solve this issue, we tried to explore this meta-analysis and comprehensively systematic review.

Our study results show that DFS observed after CS was significantly lower than that after RP; however, only an insignificant negative was observed compared with RT. More importantly, when parameters of pooled OS, DSS, rates of biopsy positivity and metastasis were evaluated, CS had more satisfactory tendencies, which implied support for its usage regardless of primary or salvage treatment. Furthermore, differences were insignificant for all primary outcomes in sensitivity analysis compared with RT. Similarly, relatively satisfactory results could be achieved from the systematic reviews of databases of large samples and single-arm studies^49^, although worse results were seemingly revealed in salvage treatments compared with primary procedures.

On the other hand, Simoneau believed that complication rates of CS were acceptable and major complications of CS included urethral sloughing (leading to retention), rectal fistula, incontinence and erectile dysfunction^50^. However, pooled data in our analysis showed more complications in the CS group compared with the RT group, in both urinary and sexual bothers. In addition, a relatively large proportion of patients from the systematic review had complications. Furthermore, significantly decreased scores according to UCLA-PCI were also dynamically shown by Malcolm *et al*.^51^, but these results were unavailable for pooling analysis. However, such adverse events are being decreased with the evolution of new technologies, including penile rehabilitation with a vacuum therapy device^52^. Furthermore, salvage CS may be associated with a higher frequency of both urinary and sexual functions than primary CS^53^, however, this finding could not be validated in our study.

With regard to the choice of primary CS, materials from propensity-weighted analyses revealed that older patients, having one comorbidity, low income, a diagnosis of indolent cancer and lower Gleason scores/D’Amico risk could be more likely to choose CS as an initial treatment^17^. Moreover, Nomura *et al*. thought CS could be used in any tumor grade of PCa with clinical stages T1c-T3, in which primary CS is suitable for low-risk patients whereas effective for the intermediate-risk patients^54^.

Furthermore, Mouraviev *et al*. concluded that CS should be recognized as an established salvage therapeutic option, and patients with serum PSA of <10 ng/ml, Gleason score of ≤8 and clinical stage of T1c or T2 before therapy are suitable for salvage CS followed by RT^55^. Similar beneficial outcomes of salvage CS compared with salvage androgen deprivation therapy were obtained in another RCT^56^. In Han’s opinion, CS was indicated as an alternative to RP or RT in low-risk patients, as primary therapy in patients with higher surgical risk, and as a salvage procedure in patients who had not responded to RT^57^. However, detailed stratifications based on these factors were not presented in our study, which would be its biggest limitation; however, this problem could not have been avoided in the present study.

In our study, seven studies had focused on focal CS while nine studies on whole-gland CS for PCa, which revealed comparable results, even worse results for whole-gland treatment in part of studies, for primary outcomes. However, trends of more patients with advanced PCa could be found in studies using whole-gland CS in further analysis. In theory, whole-gland ablation might capture more favorable survival compared to focal therapy for similar PCa patients as more cancer demised, while more complications must take into consideration. In an age-matched comparison between focal and whole-gland procedures for primary low-risk PCa patents^58^, comparable 5-year survival and higher erectile function preservation rate could be found in focal therapy group. However, comparison of these two methods for more advanced patients and salvage option need to be further discussed.

Almost half of the publications cited in our study reported the efficacy of primary CS for clinically localized PCa, which revealed overall DFS percentages of 44.6–100%. However, whether recurrence patients experiencing primary CS need to be treated by salvage methods remain unclear except for Chang’s study^44^. Except for repeat CS, other available options for recurrence include EBRT, RP, endocrine therapy and watchful waiting, of which EBRT would to be the most reasonable choice.

Finally, several important limitations, which might influence the reliability of our results, must be taken into consideration. First, probably because of the relative novelty and limited usage of CS, the pooling results cannot be generalized, considering that only two RCTs and a few comparative studies had focused on CS vs RT/RP. Second, as mentioned above, the stratification factors of PCa, such as PSA level, Gleason score, D’Amico risk classification, clinical stage, was complex, making it very hard for more detailed analysis apart from meta-analysis. Furthermore, because EBRT and BT were merged into the RT group in the meta-analysis, bias could have been generated to a great or lesser extent. Similarly, the results of meta-analysis should be carefully cautioned as large heterogeneity existed, which might be caused by differences between patients, operators, etc. However, to some degree, limitations from these aspects could be supplemented by systematic review. Nonetheless, more accurate results are still urgently required.

In summary, pooling data based on meta-analysis and databases supports the conclusion that CS would be a relatively efficient minimally invasive choice for clinically localized PCa with no significant lower OS, DSS and DFS compared with RT and RP. Similarly favorable results could be also supported in some degree by the systematic review for primary and salvage treatments. However, the large number of adverse events in urinary and genital systems indicates that clinicians should exercise caution and further prove its safety. In future, a more advanced operating system for CS with a lower occurrence of complications is expected. More importantly, more well-designed RCTs and high-quality prospectively comparative studies with long-term follow-up results should be conducted to accurately solve this issue and complete our meta-analysis.

## Methods

### Literature-search strategy

In March 2016, two reviewers independently carried out a systematic search of three databases—PubMed, Embase and the Cochrane Library for all English literature published on or before December 15 without of any restrictions. The following MeSH terms and their combinations were searched to identify relevant studies in [Title/Abstract]: prostate/prostatic, cancer/carcinoma/adenocarcinoma, cryoablation/cryosurgery/cryotherapy/cryotreatment.

### Inclusion and exclusion criteria

All available randomized controlled trials (RCTs) and controlled studies (prospective or retrospective) which had compared primary or salvage CS with RT (external beam radiation therapy [EBRT]/brachytherapy [BT]) or RP (through open/laparoscopic/robot-assisted approaches) in patients with clinically localized PCa were included in the review. In addition, materials reporting results of CS in single-arm trials were also reviewed systematically. To supplement these data, the related reference lists from identified documents were also acquired, and all computer searches were supplemented with a manual search. When multiple reports described the same population, the most comprehensive or recent was used.

Patients with organ metastasis were excluded. Because of the possibly unavoidable replication with small-scale studies, patients from registered databases were excluded from meta-analysis but were included in the supplementary systematic review. Finally, data from conference abstracts, papers that were not extractable or whose data were not available for our analyses were also disregarded.

### Data extraction and outcomes of interest

Data were extracted from the studies and compiled by two reviewers. In the case of any disagreement, a consensus was reached by Yuan after a discussion. Moreover, in the case of studies dividing patients into more than three groups, comparisons between CS and RP or RT were extracted, in which the RP group, including open/laparoscopy/robot-assisted approach, and the RT group, with EBRT/BT, were included. Meanwhile, patients who had experienced total or subtotal CS were included in the CS group. The levels of evidence of all controlled studies were evaluated using the criteria from the Centre for Evidence-Based Medicine in Oxford. However, the blinding method was not analyzed in this review because of its unsuitability for clinical trials of surgical methods.

The primary outcomes were overall survival (OS), disease-specific survival (DSS) and disease-free survival (DFS) of each intervention. The secondary events were complications caused by procedures and obtained through questionnaires, which predominantly included urinary and sexual bothers, mainly erectile dysfunction (ED). Continuous results of urinary and sexual bother scores were recorded according to the University of California Los Angeles, Prostate Cancer Index (UCLA PCI), which measures disease-specific health-related quality of life in six domains: sexual function and sexual bother, urinary function and urinary bother, and bowel function and bowel bother, whereas dichotomous variables were graded according to patients’ reporting of moderate to severe urinary and sexual bothers. After summarizing the finding, the rates of biopsy positivity and metastasis were also pooled as secondary outcomes.

### Quality assessment and statistical analysis

All the meta-analyses were performed using Review Manager 5.2 (Cochrane Collaboration, Oxford, UK). The weighted mean difference (WMD) and risk ratio (RR) were used to describe results for continuous and dichotomous variables, respectively. For studies that presented medians of follow-up results, the approximate mean was used. All results were reported with 95% confidence intervals (CIs).

Meta-analysis was performed using the random-effects method or the fixed-effects method if significant heterogeneity was not observed. Statistical heterogeneity between trials was evaluated using the I^2^ and chi-square tests with significance set as p values of <0.10. I^2^ value of 25%, 50% and 75% corresponded to low, medium and high levels of heterogeneity, respectively.

## Additional Information

**How to cite this article**: Gao, L. *et al*. Cryosurgery would be An Effective Option for Clinically Localized Prostate Cancer: A Meta-analysis and Systematic Review. *Sci. Rep*. **6**, 27490; doi: 10.1038/srep27490 (2016).

## Supplementary Material

Supplementary Information

## Figures and Tables

**Table 1:** Basic information and characteristics of studies for meta-analysis.

Author	Year	Design	Level of evidence	Patients		Freeze-thaw cycle(s)	Mean follow up (Months)	Radiation dose/ Surgical mode	Duration of ADT*	Matching	Definition of recurrence	Primary or salvage
				CS	RT/RP							
CS vs RT												
Ball *et al*.^5^	2006	P	2a	39	118	Double	6	45 Gy EBRT + 100 Gy BT or 125 Gy BT (Pd -103)	NA	1, 4, 5, 6, 8	NA	Both
Hubosky *et al*.^6^	2007	R	3b	63	63	Double	12.7	125 Gy BT (Pd -103) or EBRT + BT	NA	1, 2, 3, 4, 5, 6, 7, 8	PSA > 0.4 ng/ml or 3 consecutive rises in PSA	Primary
Chin *et al*.^7^	2008	RCT	1b	33	31	Double	37	66 Gy EBRT	3 months pre- and 3 months post- treatment	1, 2, 4, 5, 6, , 8, 9	Nadir +2 ng/ml	Primary
Chin *et al*.^8^	2012			31	31		105.2					
Robinson *et al*.^9^	2009	RCT	1b	117	114	Double	100	<70/70/73.5 Gy EBRT	3/6 months pretreatment	1, 2, 4, 5, 6, 7, 8, 9	PSA > 0.1 ng/ml or nadir +2 ng/ml	Primary
Donnelly *et al*.^10^												
Peters *et al*.^11^	2013	R	3a	54	31	Double	36 vs 108	145 Gy BT (I-125)	3 months pretreatment	1, 2, 3, 4, 5, 6, 8, 9	PSA > 0.1 ng/ml or nadir +2 ng/ml	Salvage
CS vs RP												
Gould^12^	1999	R	3b	76	83	NA	25	Open	NA	1, 3, 4	PSA > 0.2 ng/ml	Primary
Pisters *et al*.^13^	2009	R	3b	56	42	Single and double	66 vs 93.6	NA	NA	1, 2, 4, 5, 6	PSA > 0.4 ng/ml or nadir +2 ng/ml	Salvage
Peters *et al*.^11^	2013	R	3a	54	44	Double	36 vs 60	Open	≥3 months. pretreatment	1, 2, 3, 4, 5, 6, 8, 9	PSA > 0.1 ng/ml or nadir +2 ng/ml	Salvage
Elkjær *et al*.^14^	2014	P	3b	40	350	Double	29.5 vs 37	Open and robot	NA	1, 2, 3, 4, 5, 6, 7	PSA > 0.2 ng/ml or nadir +2 ng/ml	Primary

Matching: 1 = age; 2 = inclusion and exclusion criteria; 3 = stratification; 4 = PSA level; 5 = biopsy Gleason score; 6 = clinical stage; 7 = D’Amico risk group; 8 = adjuvant deprivation therapy (ADT) before treatment; 9 =ADT after treatment.

^*^Not all patients had experienced adjuvant deprivation treatment (ADT) for studies have reported ADT. NA: not available; CS: cryosurgery; RT: radiotherapy; RP: radical prostatectomy; R: retrospective; P: prospective; RCT: randomized controlled trial; EBRT: external beam radiation therapy; BT: brachytherapy; PSA: prostate specific antigen; Pd: palladium; I: iodine.

**Table 2:** Basic information, characteristics and outcomes of comparative studies using materials from registry databases.

Author	Year	Database	Design	Level of evidence				Matching			Mean follow-up (months)	Complications (%)						
												Patients			Results (%)		Overall		Urinary			Sexual	
					CS	RT	RP		OS	DSS							CS	RT	CS	RT	RP	CS	RT
White *et al*.^15^	2008	CaPSURE	R	3b	12	26	31	1, 2, 4, 6, 8, 9	–	–	51.4	Dynamically changes at years of 1, 2 and 3 according to UCLA PCI score						
Williams *et al*.^16^	2011	SEER	P	2a	943	9985	–	1, 2, 4, 6, 8, 10	–	–	>36	65.0	48.8	39.2	22.2	–	35.1	21.1
Roberts *et al*.^17^	2012	SEER	R	2b	380	10757	3960	1, 2, 4, 6, 7, 8, 9	–	–	>12	–	–	>28.7	–	–	20.1	–
Friedlander *et al*.^18^	2014	SEER	R	2b	341	–	99	1, 2, 5, 8, 10	93.9 vs 78.4	98.6 vs 93.5	15 vs 30	–	–	–	–	–	–	–
Jarosek *et al*.^19^	2014	SEER	P	2a	2115	70412	26790	1, 2, 3, 4, 8	–	–	49.7	–	–	19.4	19.7	27.2	–	–

Matching: 1 = age; 2 = inclusion and exclusion criteria; 3 = matched design; 4 = primary treatment; 5 = salvage treatment; 6 = PSA level; 7 = biopsy Gleason score; 8 = clinical stage; 9 = D’Amico risk group; 10 = adjuvant deprivation therapy (ADT); CaPSURE: the Cancer of the Prostate Strategic Research Endeavor registry; SEER: Surveillance Epidemiology and End Results-Medicare linked database; OS: overall survival; DSS: disease specific survival; UCLA PCI: the University of California Los Angeles, Prostate Cancer Index.

**Table 3:** Basic information, characteristics and outcomes from single-arm studies.

Author	Year	Design	Primary or salvage	Patients	Basic information	Results (%)			Mean follow-up (months)	Complications (%)		
						OS	DSS	bDFS		Overall	Urinary	ED
Pisters *et al*.^20^	1999	R	Salvage	145	5, 6, 7, 8, 10	–	–	55.2	>6	–	–	–
De La Taille *et al*.^21^	2000	P	Both	35	1, 2, 5, 6, 7, 10	100	100	70(9 months)	8.3	–	>8.6	–
Izawa *et al*.^22^	2002	R	Salvage	131	5, 6, 7, 8,	73(5 yrs)	79(5 yrs)	40(5 yrs)	57.6	–		
Bahn *et al*.^23^	2002	R	Primary	590	1, 2, 5, 6, 7, 8, 10		–	89.5(7 yrs)	65.2	–	>15.9	56.8 increased
Prepelica *et al*.^24^	2005	R	Primary	65	1, 2, 5, 6, 7, 9, 10	100	100	83.3	35	–	–	–
Bahn *et al*.^25^	2006	R	Primary	31	1, 2, 6, 7	96.8	100	92.9	70	–	–	11.1(potency preservation)
Ellis *et al*.^26^	2007	NA	Primary	416	1, 5, 6, 7, 8, 9	–	–	79.6(4 yrs)	20.4	–	–	–
Ismail *et al*.^27^	2007	P	salvage	100	1, 2, 5, 6, 7, 8, 9, 10	–	100	83%(1 yr); 72%(2 yrs); 59%(3 yrs); 25%(5 yrs)	33.5	–	>16	86
Ng *et al*.^28^	2007	R	Salvage	187	1, 5, 6, 7, 8, 10	97 (5 yrs); 92 (8 yrs)	–	51	39	–	>39	–
Diblasio *et al*.^29^	2008	R	Primary	78	1, 3, 4, 5, 6, 7, 8, 10	95.9%(1 yr); 94.3(3 yrs); 94.3(5 yrs)	100	97.9%(1 yr); 82.9%(3 yrs); 82.9(5 yrs)	39.8	–	41 increased	33.4 increased for erectile function of 3 level and higher
Jones *et al*.^30^	2008	R	Primary	77	1, 2, 6, 7	–	–	44.6	39.0	–	–	–
Truesdale *et al*.^31^	2010	R	Primary	77	1,4, 6, 7, 8, 9,	–	–	72.7	24	–	Decreased scores according to AUA SI and IIEF-5, respectively	
Williams *et al*.^32^	2011	R	Salvage	176	1, 5, 6, 7, 8, 10	95(5 yrs); 91(8 yrs); 87(10 yrs)	–	47(5 yrs); 39(8 yrs); 39(10 yrs)	89.5	–	–	–
Caso *et al*.^33^	2012	R	Primary	97	1, 2, 4, 5, 6, 7, 8	–	–	Biopsy: 87.6	48	–	–	–
Kim *et al*.^34^	2012	R	Primary	10	1, 6, 7, 9	100	100	100	13	–	>30	–
Castro Abreu *et al*.^35^	2013	R	Salvage	50	1, 6, 7, 8, 10	100	100	78 (5 yrs)	>31	–	–	–
Durand *et al*.^36^	2013	P	Primary	48	1, 2, 3, 6, 7, 8, 9, 10	100	100	Biopsy: 84	13.2	19	No significantdecreased	Significant decrease in 3 months but in 6 months
Al Ekish *et al*.^37^	2013	R	Both	30	1, 2, 6, 7, 9, 10	100	100	93.3	18	–	–	–
Hale *et al*.^38^	2013	R	Primary	26	1, 2, 4, 6, 7, 8	100	100	88.5	19.1	–	–	27 increased
Ullal *et al*.^39^	2013	R	Both	32	1, 6, 7	–	–	68.8	41.2	–	–	–
Li *et al*.^40^	2014	R	Salvage	91	1, 2, 6, 7, 8, 10	98.9(5 yrs)	–	95.3(1 yr); 72.4(3 yrs); 46.5(5 yrs)	15	–	>6.6	11.0 increased
Guo *et al*.^41^	2014	R	Primary	75	1, 2, 5, 6, 7, 8, 9	85.3(5 yrs)	92.0(5 yrs)	48(5 yrs)	75	–	–	–
Rodríguez *et al*.^42^	2014	P	Primary	102	1, 5, 6, 7, 8, 9, 10	94.4	98.1	76.9	61	>11.1	>5.6	32.1 increased
Kvorning Ternov *et al*.^43^	2014	R	Salvage	30	1, 2, 6, 7, 8, 9, 10	93.3	96.7	43.5	32.4	16.7 for grade 3 and 4	>53.3	100
Chang *et al*.^44^	2014	R	Salvage	12	1, 2, 6, 7, 8, 10	–	100	58.3	33	–	16.7	16.7
Elshafei *et al*.^45^	2015	R	Primary	2242	1, 2, 4, 5, 7, 8, 9	–	–	72.8(5yrs)	32.6	–	–	–
Tay *et al*.^46^	2016	R	Primary	300	1, 2, 6, 7, 8, 9, 10	96.7	–	77.2(2yrs), 59.1(5yrs)	28.4	–	>9.7	83.5
Lian *et al*.^47^	2016	R	Primary	40	1, 2, 5, 6, 7, 8, 9, 10	100	100	95	63	–	–	36.6

Matching: 1 = age; 2 = inclusion and exclusion criteria; 3 = body mass index (BMI); 4 = race; 5 = stratification; 6 = PSA level; 7 = biopsy Gleason score; 8 = clinical stage; 9 = D’Amico risk group; 10 = adjuvant deprivation therapy (ADT); bDFS: biochemical disease free survival; ED: erectile dysfunction; AUA SI: American Urological Association Symptom Index; IIEF: International Index of Erectile Function; IPSS: International Prostate Symptom Score; yrs: years.

## References

[b1] Siegel R., Ma J., Zou Z. & Jemal A. Cancer statistics. CA-Cancer J Clin. 64, 9–29 (2014). 24399786 10.3322/caac.21208

[b2] Wong Y. N. . Survival associated with treatment vs observation of localized prostate cancer in elderly men. Jama. 296, 2683–2693 (2006). 17164454 10.1001/jama.296.22.2683

[b3] Langenhuijsen J. F., Broers E. M. & Vergunst H. Cryosurgery for prostate cancer: an update on clinical results of modern cryotechnology. Eur Urol. 55, 76–86 (2009). 18789572 10.1016/j.eururo.2008.08.063

[b4] Baust J. G. & Gage A. A. The molecular basis of cryosurgery. BJU Int. 95, 1187–1191 (2005). 15892798 10.1111/j.1464-410X.2005.05502.x

[b5] Ball A. J. . Fourth Prize: Prospective Longitudinal Comparative Study of Early Health-Related Quality-of-Life Outcomes in Patients Undergoing Surgical Treatment for Localized Prostate Cancer: A Short-Term Evaluation of Five Approaches from a Single Institution. J Endourol. 20, 723–731 (2006). 17094746 10.1089/end.2006.20.723

[b6] Hubosky S. G. . Single center experience with third-generation cryosurgery for management of organ-confined prostate cancer: critical evaluation of short-term outcomes, complications, and patient quality of life. J Endourol. 21, 1521–1532 (2007). 18186694 10.1089/end.2007.9875

[b7] Chin J. . Randomized trial comparing cryoablation and external beam radiotherapy for T2C-T3B prostate cancer. Prostate Cancer P D. 11, 40–45 (2007). 10.1038/sj.pcan.4500988 17579613

[b8] Chin J. L., Al-Zahrani A. A., Autran-Gomez A. M., Williams A. K. & Bauman G. Extended followup oncologic outcome of randomized trial between cryoablation and external beam therapy for locally advanced prostate cancer (T2c-T3b). J Urol. 188, 1170–1175 (2012). 22901586 10.1016/j.juro.2012.06.014

[b9] Robinson J. W. . A randomized trial of external beam radiotherapy versus cryoablation in patients with localized prostate cancer: quality of life outcomes. Cancer. 115, 4695–4704 (2009). 19691092 10.1002/cncr.24523

[b10] Donnelly B. J. . A randomized trial of external beam radiotherapy versus cryoablation in patients with localized prostate cancer. Cancer. 116, 323–330 (2010). 19937954 10.1002/cncr.24779

[b11] Peters M. . Patterns of outcome and toxicity after salvage prostatectomy, salvage cryosurgery and salvage brachytherapy for prostate cancer recurrences after radiation therapy: a multi-center experience and literature review. World J Urol. 31, 403–409 (2013). 22903773 10.1007/s00345-012-0928-8

[b12] Gould R. S. Total cryosurgery of the prostate versus standard cryosurgery versus radical prostatectomy: comparison of early results and the role of transurethral resection in cryosurgery. J Urol. 162, 1653–1657 (1999). 10524891

[b13] Pisters L. L. . Locally recurrent prostate cancer after initial radiation therapy: a comparison of salvage radical prostatectomy versus cryotherapy. J Urol. 182, 517–527 (2009). 19524984 10.1016/j.juro.2009.04.006

[b14] Elkjær M. C. & Borre M. Oncological outcome after primary prostate cryoablation compared with radical prostatectomy: A single-centre experience. Scand J Urol. 48, 27–33 (2014). 23597178 10.3109/21681805.2013.792102

[b15] White W. M., Sadetsky N., Waters W. B., Carroll P. R. & Litwin M. S. Quality of life in men with locally advanced adenocarcinoma of the prostate: an exploratory analysis using data from the CaPSURE database. J Urol. 180, 2409–2414 (2008). 18930270 10.1016/j.juro.2008.08.079

[b16] Williams S. B. . Comparative effectiveness of cryotherapy vs brachytherapy for localised prostate cancer. BJU Int. 110, E92–E98 (2012). 22192688 10.1111/j.1464-410X.2011.10775.x

[b17] Roberts C. B. . Treatment profile and complications associated with cryotherapy for localized prostate cancer: a population-based study. Prostate Cancer P D. 14, 313–319 (2011). 10.1038/pcan.2011.17 PMC3151329 21519347

[b18] Friedlander D. F. . Population-based Comparative Effectiveness of Salvage Radical Prostatectomy vs Cryotherapy. Urology. 83, 653–657 (2014). 24581527 10.1016/j.urology.2013.11.019

[b19] Jarosek S. L., Virnig B. A., Chu H. & Elliott S. P. Propensity-weighted Long-term Risk of Urinary Adverse Events After Prostate Cancer Surgery, Radiation, or Both. Eur Urol. 67, 273–280 (2014). 25217421 10.1016/j.eururo.2014.08.061

[b20] Pisters L. L., Perrotte P., Scott S. M., Greene G. F. & von Eschenbach A. C. Patient selection for salvage cryotherapy for locally recurrent prostate cancer after radiation therapy. J Clin Oncol. 17, 2514–2514 (1999). 10561317 10.1200/JCO.1999.17.8.2514

[b21] De La Taille A. . Cryoablation for clinically localized prostate cancer using an argon‐based system: complication rates and biochemical recurrence. BJU Int. 85, 281–286 (2000). 10671882 10.1046/j.1464-410x.2000.00456.x

[b22] Izawa J. I. . Salvage cryotherapy for recurrent prostate cancer after radiotherapy: variables affecting patient outcome. J Clin Oncol. 20, 2664–2671 (2002). 12039928 10.1200/JCO.2002.06.086

[b23] Bahn D. K. . Targeted cryoablation of the prostate: 7-year outcomes in the primary treatment of prostate cancer. Urology. 60, 3–11 (2002). 12206842 10.1016/s0090-4295(02)01678-3

[b24] Prepelica K. L., Okeke Z., Murphy A. & Katz A. E. Cryosurgical ablation of the prostate: high risk patient outcomes. Cancer. 103, 1625–1630 (2005). 15747374 10.1002/cncr.20944

[b25] Bahn D. K. . Focal prostate cryoablation: initial results show cancer control and potency preservation. J Endourol. 20, 688–692 (2006). 16999628 10.1089/end.2006.20.688

[b26] Ellis D. S., Manny T. B. Jr & Rewcastle J. C. Cryoablation as primary treatment for localized prostate cancer followed by penile rehabilitation. Urology. 69, 306–310 (2007). 17320669 10.1016/j.urology.2006.10.024

[b27] Ismail M., Ahmed S., Kastner C. & Davies J. Salvage cryotherapy for recurrent prostate cancer after radiation failure: a prospective case series of the first 100 patients. BJU Int. 100, 760–764 (2007). 17662081 10.1111/j.1464-410X.2007.07045.x

[b28] Ng C. K., Moussa M., Downey D. B. & Chin J. L. Salvage cryoablation of the prostate: followup and analysis of predictive factors for outcome. J Urol. 178, 1253–1257 (2007). 17698104 10.1016/j.juro.2007.05.137

[b29] Diblasio C. J. . Contemporary analysis of erectile, voiding, and oncologic outcomes following primary targeted cryoablation of the prostate for clinically localized prostate cancer. Int Braz J Urol 34, 443–450 (2008). 18778495 10.1590/s1677-55382008000400006

[b30] Jones J. S. & Rewcastle J. C. Primary cryoablation for Gleason 8, 9, or 10 localized prostate cancer: Biochemical and local control outcomes from the Cryo OnLine database registry. Indian J Urol. 24, 490–493 (2008). 19468503 10.4103/0970-1591.44254 PMC2684392

[b31] Truesdale M. D. . An evaluation of patient selection criteria on predicting progression-free survival after primary focal unilateral nerve-sparing cryoablation for prostate cancer: recommendations for follow up. Cancer J. 16, 544–549 (2010). 20890154 10.1097/PPO.0b013e3181f84639

[b32] Williams A. K. . Disease-free survival following salvage cryotherapy for biopsy-proven radio-recurrent prostate cancer. Eur Urol. 60, 405–410 (2011). 21185115 10.1016/j.eururo.2010.12.012

[b33] Caso J. R., Tsivian M., Mouraviev V. & Polascik T. J. Predicting biopsy-proven prostate cancer recurrence following cryosurgery. Urol Oncol. 30, 391–395 (2012). 20826095 10.1016/j.urolonc.2010.04.001

[b34] Kim F. J. . Initial Brazilian experience in the treatment of localized prostate cancer using a new generation cryotechnology: feasibility study. Int Braz J Urol. 38, 620–626 (2012). 23131504 10.1590/s1677-55382012000500006

[b35] Castro Abreu A. L. . Salvage focal and salvage total cryoablation for locally recurrent prostate cancer after primary radiation therapy. BJU Int. 112, 298–307 (2013). 23826840 10.1111/bju.12151

[b36] Durand M. . Focal cryoablation: a treatment option for unilateral low‐risk prostate cancer. BJU Int. 113, 56–64 (2014). 24053685 10.1111/bju.12370

[b37] Al Ekish S., Nayeemuddin M., Maddox M. & Pareek G. The role of cryosurgery of the prostate for nonsurgical candidates. JSLS. 17, 423–428 (2013). 24018080 10.4293/108680813X13693422518551 PMC3771762

[b38] Hale Z., Miyake M., Palacios D. A. & Rosser C. J. Focal cryosurgical ablation of the prostate: a single institute’s perspective. BMC Urol. 13, 2, doi: 10.1186/1471-2490-13-2 (2013). 23311921 PMC3585847

[b39] Ullal A. V., Korets R., Katz A. E. & Wenske S. A Report on Major Complications and Biochemical Recurrence After Primary and Salvage Cryosurgery for Prostate Cancer in Patients With Prior Resection for Benign Prostatic Hyperplasia: A Single-center Experience. Urology. 82, 648–652 (2013). 23831070 10.1016/j.urology.2013.04.052

[b40] Li Y. H. . Salvage focal prostate cryoablation for locally recurrent prostate cancer after radiotherapy: Initial results from the cryo on-line data registry. Prostate. 75, 1–7 (2015). 25283814 10.1002/pros.22881

[b41] Guo Z., Si T., Yang X. & Xu Y. Oncologic Outcomes of Cryosurgery as Primary Treatment in T3 Prostate Cancer: Experience of a Single Center. BJU Int. 116, 79–84 (2014). 25168692 10.1111/bju.12914

[b42] Rodríguez S. A. . Cryotherapy for Primary Treatment of Prostate Cancer: Intermediate Term Results of a Prospective Study from a Single Institution. Prostate Cancer. 2014, 571576, doi: 10.1155/2014/571576 (2014). 24693437 PMC3945790

[b43] Kvorning Ternov K., Krag Jakobsen A., Bratt O. & Ahlgren G. Salvage cryotherapy for local recurrence after radiotherapy for prostate cancer. Scand J Urol. 49, 115–119 (2014). 25428754 10.3109/21681805.2014.968869

[b44] Chang X. . Salvage cryosurgery for locally recurrent prostate cancer after primary cryotherapy. Int Urol Nephrol. 47, 301–305 (2015). 25510358 10.1007/s11255-014-0887-7

[b45] Elshafei A. . A Pretreatment Nomogram for Prediction of Biochemical Failure After Primary Cryoablation of the Prostate. Prostate. 75, 1447–1453 (2015). 26172607 10.1002/pros.23030

[b46] Tay K. J. . Primary Cryotherapy for High-Grade Clinically Localized Prostate Cancer: Oncologic and Functional Outcomes from the COLD Registry. J Endourol. 30, 43–48 (2016). 26414656 10.1089/end.2015.0403

[b47] Lian H. . Focal cryoablation for unilateral low–intermediate–risk prostate cancer: 63-month mean follow–up results of 41 patients. Int Urol Nephrol. 48, 85–90 (2016). 26531063 10.1007/s11255-015-1140-8

[b48] Hoffmann N. E. & Bischof J. C. The cryobiology of cryosurgical injury. Urology 60, 40–49 (2002). 12206847 10.1016/s0090-4295(02)01683-7

[b49] Chin J. L., Lim D. & Abdelhady M. Review of primary and salvage cryoablation for prostate cancer. Cancer Control. 14, 231–237 (2007). 17615528 10.1177/107327480701400305

[b50] Simoneau A. R. Treatment-and disease-related complications of prostate cancer. Rev Urol. 8, S56–S67 (2006). 17021643 PMC1578722

[b51] Malcolm J. B. . Quality of life after open or robotic prostatectomy, cryoablation or brachytherapy for localized prostate cancer. J Urol. 183, 1822–1829 (2010). 20303100 10.1016/j.juro.2009.12.102

[b52] Ellis D. S., Manny T. B. Jr & Rewcastle J. C. Focal cryosurgery followed by penile rehabilitation as primary treatment for localized prostate cancer: initial results. Urology 70, S9–S15 (2007). 10.1016/j.urology.2007.07.036 18194712

[b53] Anastasiadis A. G. . Comparison of health-related quality of life and prostate-associated symptoms after primary and salvage cryotherapy for prostate cancer. J Cancer Res Clin Oncol. 129, 676–682 (2003). 14569465 10.1007/s00432-003-0472-4 PMC12161918

[b54] Nomura T. & Mimata H. Focal therapy in the management of prostate cancer: an emerging approach for localized prostate cancer. Adv Urol. 2012, 391437, doi: 10.1155/2012/391437 (2012). 22593764 PMC3347714

[b55] Mouraviev V., Spiess P. E. & Jones J. S. Salvage cryoablation for locally recurrent prostate cancer following primary radiotherapy. Eur Urol. 61, 1204–1211 (2012). 22421081 10.1016/j.eururo.2012.02.051

[b56] Salji M. . Feasibility study of a randomised controlled trial to compare (deferred) androgen deprivation therapy and cryotherapy in men with localised radiation-recurrent prostate cancer. Br J Cancer 111, 424–429 (2014). 24946001 10.1038/bjc.2014.316 PMC4119985

[b57] Han K.-R. . Treatment of organ confined prostate cancer with third generation cryosurgery: preliminary multicenter experience. J Urol. 170, 1126–1130 (2003). 14501706 10.1097/01.ju.0000087860.52991.a8

[b58] Mendez M. H., Passoni N. M., Pow-Sang J., Jones J. S. & Polascik T. J. Comparison of Outcomes Between Preoperatively Potent Men Treated with Focal Versus Whole Gland Cryotherapy in a Matched Population. J Endourol. 29, 1193–1198 (2015). 26058496 10.1089/end.2014.0881

