# Supplementary material for: Cryosurgery would be An Effective Option for Clinically Localized Prostate Cancer: A Meta-analysis and Systematic Review
Source: Sci Rep. 2016 Jun 7;6:27490. doi: 10.1038/srep27490 (PMC4895342; doi:10.1038/srep27490)
Supplement: Supplementary Information [file srep27490-s1.pdf]

Cryosurgery would be An Effective Option for Clinically Localized Prostate Cancer: A Meta-analysis and Systematic Review

Liang Gao†, Lu Yang†, Shengqiang Qian, Zhuang Tang, Feng Qin, Qiang Wei, Ping Han, Jiuhong Yuan

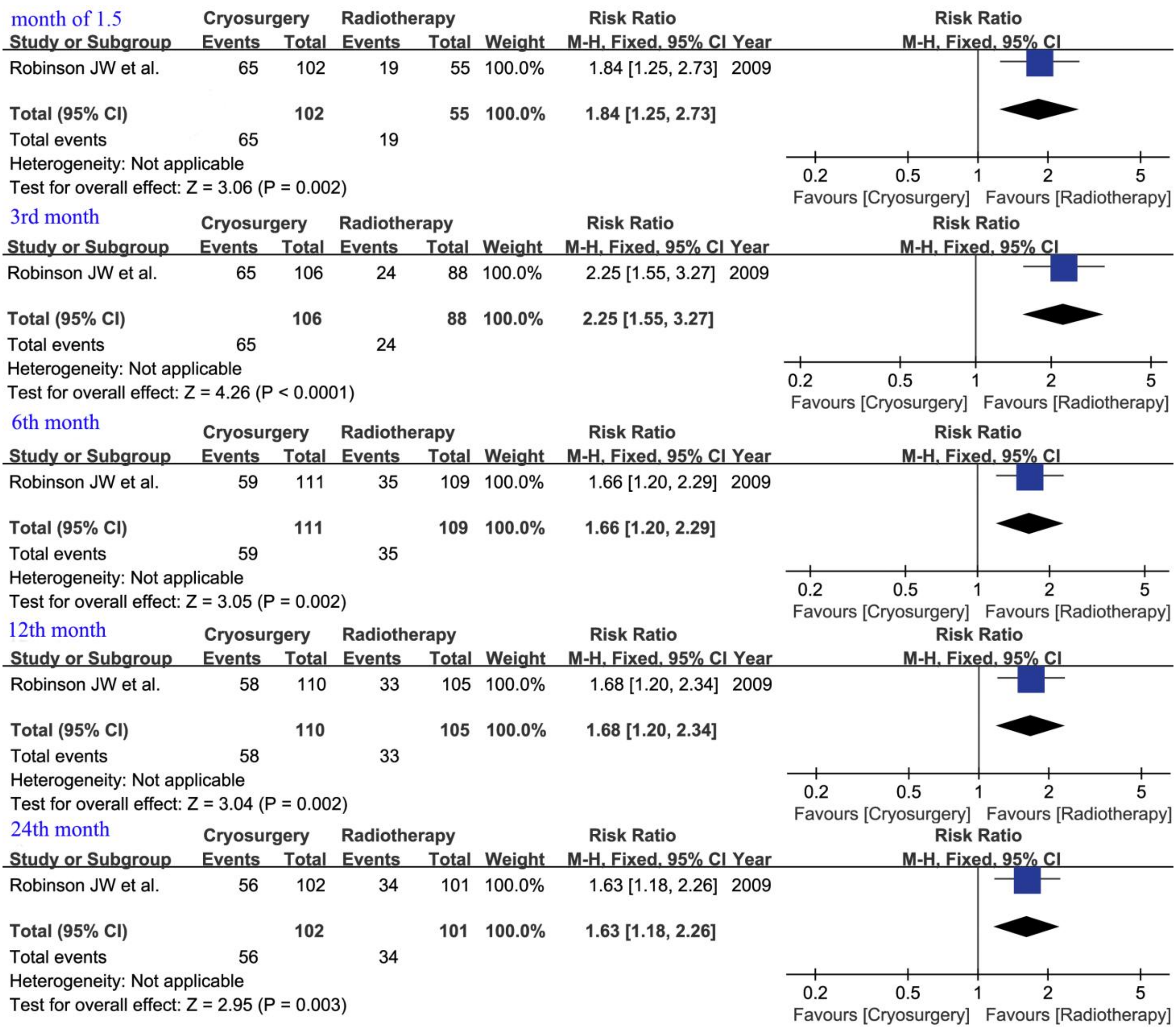

Figure. Dynamical forest plot and meta-analysis of patients reporting a moderate or severe problem of sexual bother in comparisons of CS vs RT in months of 1.5, 3, 6, 12 and 24, respectively.
